# Peroxisome proliferator–activated receptor delta and liver diseases

**DOI:** 10.1097/HC9.0000000000000646

**Published:** 2025-02-03

**Authors:** Tomoo Yamazaki, Edward E. Cable, Bernd Schnabl

**Affiliations:** 1 Department of Medicine, University of California San Diego, La Jolla, California, USA; 2 Department of Medicine, Division of Gastroenterology and Hepatology, Shinshu University School of Medicine, Matsumoto, Japan; 3 CymaBay Therapeutics, Fremont, California, USA; 4 Department of Medicine, VA San Diego Healthcare System, San Diego, California, USA

**Keywords:** elafibranor, metabolic dysfunction–associated steatohepatitis, peroxisome proliferator-activated receptor, primary biliary cholangitis, seladelpar

## Abstract

Peroxisome proliferator-activated receptors (PPARs) are nuclear receptors involved in transcriptional regulation and play an important role in many physiological and metabolic processes. Unlike PPAR-alpha and PPAR-gamma, PPAR-delta is ubiquitously expressed, and its activity is key to maintaining proper metabolic homeostasis within the liver. PPAR-delta not only regulates physiologic processes of lipid, glucose, and bile acid metabolism but also attenuates pathologic responses to alcohol metabolism, inflammation, fibrosis, and carcinogenesis, and is considered an important therapeutic target in liver diseases. Promising results have been reported in clinical trials for PPAR-delta agonists in liver disease, and the selective agonist seladelpar was recently conditionally approved in the United States as a new treatment option for primary biliary cholangitis. This review provides an overview of PPAR-delta’s function and biology in the liver, examines its kinetics and therapeutic potential across different liver diseases, and discusses the current status of clinical trials involving its agonists.

## INTRODUCTION

The liver is an important organ that performs various functions, including metabolic homeostasis, immune and inflammatory responses, storage of bioactive molecules, detoxification of endogenous and exogenous substances, and regulation of blood coagulation. Liver damage due to various causes, including viral, metabolic, autoimmune, and toxic, can lead to acute liver failure, or in the course of chronic liver damage, result in liver fibrosis, cirrhosis, and HCC.^1^,^2^,^3^

Peroxisome proliferator–activated receptors (PPARs) are nuclear receptors involved in transcriptional regulation and play an important role in many physiological and metabolic processes.^4^ Three subtypes, peroxisome proliferator–activated receptor-alpha (PPARA), peroxisome proliferator–activated receptor-delta (PPARD), and PPAR-gamma (PPARG) modify the transcription of genes involved in fatty acid metabolism, glucose metabolism, and inflammation by binding specific ligands.^5^ Ligands for PPARA, the fibrates, and PPARG agonists, the thiazolidinediones, have been used for the treatment of dyslipidemia and type 2 diabetes mellitus.^6^ Unlike PPARA and PPARG, PPARD (also called PPAR-beta) is ubiquitously expressed, and its function in vivo is not well understood.^7^,^8^ However, after the discovery of high-affinity agonists, such as GW0742 and GW501516, their association with obesity and diabetes, as well as malignancy, neurological disease, inflammation, dyslipidemia, and cardiac disease became apparent.^9^,^10^,^11^,^12^,^13^ In the liver, a central organ of metabolism, PPARD is also attracting attention as a new therapeutic target,^14^,^15^ and a series of very promising clinical trial results of its agonists have recently been reported for patients with primary biliary cholangitis (PBC).^16^,^17^ Based on these results, 2 drugs with agonistic effects on PPARD, elafibranor, and seladelpar, were conditionally approved by the Food and Drug Administration (FDA) in the United States as second-line therapy for patients with PBC who do not respond adequately to ursodeoxycholic acid (UDCA).

This review focuses mainly on PPARD, summarizing its general function in the liver, the status of clinical trials of related drugs, and its potential as a therapeutic target for liver disease.

### Biology of PPARs

The human genome contains a total of 48 nuclear receptors, and they all have 3 structural features in common, a ligand-binding domain, a hinge region, and a DNA-binding domain.^18^ PPARs are transcription factors discovered in rodents 30 years ago and belong to subfamily 1 of the nuclear hormone receptor superfamily.^19^,^20^ PPARs have an aminoterminal transactivation domain AF-1, a DNA-binding domain, and a carboxy-terminal dimerization and ligand-binding domain with a ligand-dependent transactivation function AF-2, and this structure is common to other nuclear receptor families.^21^ The binding of various lipophilic ligands, endogenous or exogenous, to PPARs allows them to interact with coactivator complexes. For their transcriptional activity to proceed, a series of processes such as binding to lipid ligands followed by heterodimer formation with different nuclear receptors (retinoid-X receptors), interaction with transcriptional coactivators, and binding to the PPAR response element in the promoter of the target gene are required.^22^,^23^ As a result, it regulates the expression of downstream genes with various physiological activities.

Although all PPARs play a major regulatory role in energy homeostasis, each subtype has different functions and locations of expression.^24^,^25^ The metabolic pathways through which each subtype acts are specific and complex, sometimes overlapping. Each PPAR is encoded by a different gene, but the interspecies sequences overlap. The human *PPARA* gene is located on chromosome 22, and the major organs of expression are the liver, heart, skeletal muscles, brown adipose tissue, intestine, and kidneys. PPARA functions as a central control sensor coordinating the transport, esterification, and oxidation of fatty acids, browning of brown adipose tissue, and energy dissipation.^5^,^26^ PPARA, which is most abundantly expressed in the liver, promotes fatty acid oxidation and heat production. PPARA stimulated by fasting induces transcription factors such as fasting-induced adipose factor and FGF21, which increases free fatty acids and ketone bodies to provide energy.^27^,^28^ PPARA regulates glycerol metabolism in the liver and activates the regulatory gene vanin-1, which reduces hepatic steatosis by suppressing inflammation and oxidative stress pathways.^29^ Regulation of bile acid metabolism is another important function of PPARA in the liver.^30^

The *PPARG* gene is located on chromosome 3, and the major organs of expression are white adipose tissue, liver, skeletal muscles, intestine, and immune cells. PPARG coordinates fatty acid transportation, lipid synthesis, adipogenesis, energy storage, thermogenesis, and glucose homeostasis.^31^,^32^

On the other hand, *PPARD*, located on chromosome 6, is ubiquitously expressed and is involved in fatty acid oxidation and glucose homeostasis.^33^ Each PPAR has a specific ligand affinity, which is interpreted by differences in the dimensions of the binding pocket and lipophilicity.^34^ The ligand-binding domain of PPARD is narrower than that of other PPARs, but its structural features allow it to bind various ligands with relatively low affinity.^35^ Its natural ligands include polyunsaturated fatty acids and their metabolites.^36^,^37^ Although many endogenous ligands have been reported, their physiological significance is still unclear.^38^

In addition to regulating energy and glucose metabolism in the liver, PPARs are involved in the suppression of inflammation, bile acid secretion, endoplasmic reticulum stress, and carcinogenesis, and are considered important factors in physiological and pathological processes in the liver.^39^

### The function of PPARD in the liver

PPARD expression is much higher in skeletal muscle than in the liver. In mouse liver, PPARD is highly expressed in cholangiocytes, hepatic stellate cells (﻿HSCs), and liver sinusoidal endothelial cells (﻿LSECs) compared with hepatocytes.^40^,^41^ According to recent analyses using single-cell and omics approaches, PPARD is expressed at low levels in all human hepatic cells.^42^,^43^ Furthermore, PPARA and PPARG showed different expression patterns by zonation, while PPARD and its target genes were equally expressed throughout the lobule.^44^

Hepatocyte-specific *Ppard* knockout mice showed a major role of PPARD in the metabolic regulation of the liver. Activation of PPARD increases fatty acid oxidation in hepatocytes and induces activation of PPARA, which also has fatty acid oxidation–activating properties, creating a synergistic effect.^12^,^45^ In this connection, 1-palmitoyl-2-oleyl-phosphatidylcholine, the endogenous ligand of PPARA, is known to be increased by PPARD.^46^ PPARD also increases plasma HDL by inducing the expression of apolipoprotein A-II and phospholipid transfer protein.^47^,^48^ Furthermore, PPARD improves insulin responsiveness and glucose metabolism in the liver. In this context, activation and overexpression of PPARD are thought to direct it toward the pentose phosphate pathway and glycogen synthesis rather than glucose production.^49^,^50^ These functions of PPARD in regulating lipid and energy metabolism and improving insulin sensitivity overlap with those of PPARA and PPARG, suggesting that the three subtypes sometimes have different targets but function complementary to each other.

In addition to its central role in metabolism, PPARD is also known to have an anti-inflammatory effect mediated by immune cells, especially macrophages. Deletion of *Ppard* in Kupffer cells (﻿KCs), liver resident macrophages, inhibited differentiation into anti-inflammatory (M2) macrophages, which are in a quiescent state, via reduced sensitivity to IL-4.^51^ Through a similar mechanism, transplantation of *Ppard*-null bone marrow into wild-type mice caused a decrease in M2 KCs, liver dysfunction, and insulin resistance,^51^ indicating that PPARD plays an important role in the regulation of inflammation. On the other hand, overexpression of *Ppard* in mouse KC ameliorates anoxia/reoxygenation-induced hepatocellular injury, suggesting that PPARD is strongly involved in KC-mediated inflammation in a specific pathological condition.^52^ The anti-inflammatory effect via downregulation of inflammatory mediators, such as TNF alpha, IL-1, and IL-6, is an overlapping function not only for PPARD but also for PPARA and PPARG.^53^

The high expression of *Ppard* in HSCs, cells known to play an important role in liver fibrosis, has already been reported.^40^ The PPARD agonist KD3010 showed strong antifibrotic effects in mouse models of liver fibrosis, although it did not exert a direct effect on HSCs.^14^ Rather, KD3010 protected hepatocytes against toxin-induced cell death by reducing reactive oxygen species production.^14^ PPARD agonist GW501516 attenuated hepatic fibrosis by reducing SMAD3 phosphorylation and p300 levels via AMP-activated protein kinase in HSCs, using mice and LX-2 cell lines.^54^ On the other hand, the PPARD agonist GW501516 has been reported to stimulate HSC proliferation and enhance fibrosis and inflammation via the p38 and Jun N-terminal kinase mitogen-activated protein kinase pathways.^55^ Therefore, the effect of PPARD agonism on fibrosis is controversial.

PPARD also plays an important role in the synthesis, metabolism, and transport of bile acids. Although the full extent of this regulatory mechanism is not fully understood, recent reports indicate that PPARD activation reduces bile acid synthesis via FGF21-dependent inhibition of cholesterol 7 alpha-hydroxylase in mouse and human hepatocytes.^15^ The potent induction of FGF21 by PPARA and the effect of its agonist on cholestasis are already known, and the linkage between PPARD and PPARA in the regulation of cholestasis is a subject for future research.^56^,^57^ Although the direct effects of PPARD on cholangiocytes are largely unknown, previous studies using primary cholangiocytes isolated from rodents have shown that PPARD interacts with LXR-β to regulate NPC1L1/ABCA1-dependent cholesterol transport from bile to cholangiocytes.^58^

Other aspects include reports of sex differences and diurnal variation in PPARD expression in the liver. Male rats have higher levels of PPARA mRNA and protein in the liver than female rats.^59^ Interestingly, as for *Ppard*, high expression is observed in female mouse livers.^60^ A recent analysis of mouse RNA sequencing confirms a similar trend, and the absence of the microbiome attenuates sexual dimorphism in the liver.^61^ All of these reports are based on rodent studies, and there is minimal knowledge of PPAR sex differences in humans. Evaluating the impact of human sex differences on PPAR isotype function in the liver is important for the development of gender-optimized therapies in the future. The circadian rhythm of PPAR subtypes in the liver is also worth noting. PPARA mRNA and protein levels follow a circadian rhythm in mice, peaking around the switch from the resting/fasting phase to the active/feeding phase, whereas PPARD peaks at the end of the active/feeding phase.^60^,^62^ Liver-specific *Ppard* knockout mice have disturbed day-night fluctuations in fatty acid synthesis and plasma fatty acid levels.^63^ As the pan-PPAR agonist bezafibrate altered circadian rhythms of feeding and locomotor behavior in mice,^64^ PPARD agonists could be applied to “reset” the dysregulated clocks in patients with liver disease.

**FIGURE 1:**
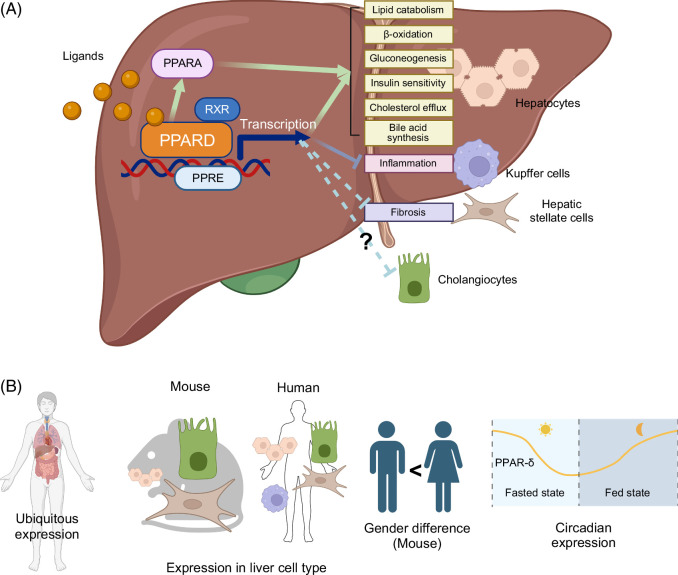
Major functions of PPARD in the liver. (A) PPARD regulates various metabolic pathways by modulating its downstream genes in the liver. It is also thought to have diverse functions, including anti-inflammatory, antifibrotic, and inhibiting cholestasis. (B) PPARD is ubiquitously expressed not only in the liver. In mouse liver, PPARD is highly expressed in cholangiocytes, HSCs, and LSECs compared with hepatocytes. On the other hand, PPARD is expressed at low levels in all human hepatic cells. Gender differences and diurnal variation in PPARD expression in the liver have been reported. Abbreviations: PPAR, peroxisome proliferator–activated receptor; PPRE, PPAR response element; RXR, retinoid X receptor.

Figure 1 summarizes the basic function of PPARD in the liver (Figure 1A) and its reported characteristics (Figure 1B).

### PPARD as a potential therapeutic target in liver diseases

#### Metabolic dysfunction–associated steatotic liver disease and Metabolic dysfunction–associated steatohepatitis

**FIGURE 2:**
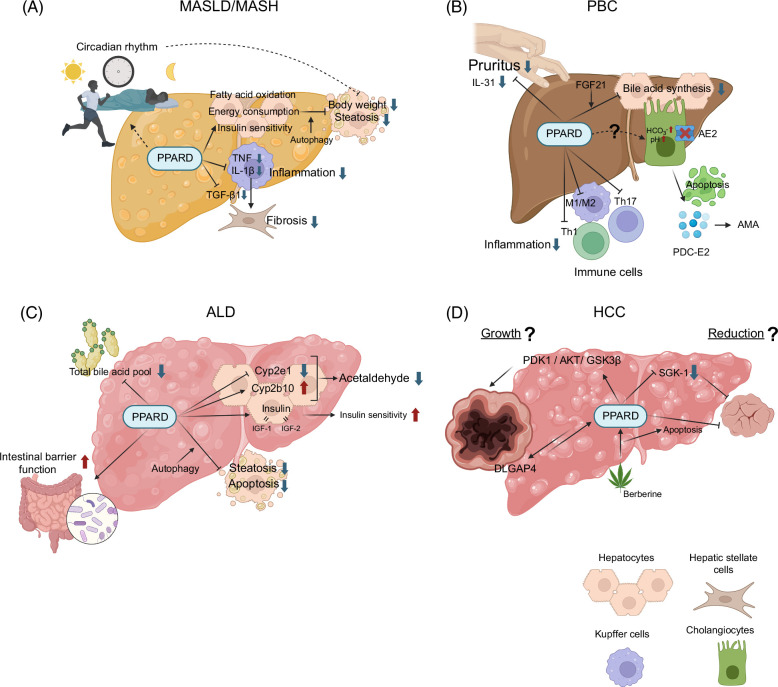
Role of﻿ PPARD for the pathogenesis of liver diseases. (A) MASLD/MASH. Activation of PPARD improves energy consumption and insulin sensitivity, leading to weight loss and liver fat reduction. Autophagy is involved in this process. PPARD activation modulates circadian rhythms of hepatic metabolism and may represent a novel therapeutic approach for MASLD/MASH. Anti-inflammatory effects via reduction of inflammatory cytokines such as TNF and IL-1β and antifibrotic effects via reduction of TGF-β1 are also expected benefits. (B) PBC. Activation of PPARD inhibits bile acid synthesis and ameliorates cholestasis, which is a hallmark in patients with PBC. This inhibitory pathway depends on the induction of FGF21. In PBC, PDC-E2 released from apoptotic cholangiocytes is presented to immune cells, resulting in the production of anti-mitochondrial antibodies. PPARD is involved in promoting differentiation into anti-inflammatory macrophages, including KCs, and in suppressing polarization of type 1 and type 17 helper T cells. The detailed mechanism of improvement in pruritus and its direct effect on cholangiocytes is not yet known. (C) Alcohol-associated liver disease. PPARD regulates cytochrome P450 enzymes that play an important role in alcohol metabolism in the liver, reducing toxic acetaldehyde. In addition, amelioration of alcohol-induced insulin resistance and amelioration of steatosis through autophagy are potential therapeutic roles of PPARD in ALD. PPARD also contributes to decreased liver and serum bile acids, and improved intestinal barrier function in ALD. (D) HCC. The function of PPARD in HCC is still under discussion, with both tumor-promoting and tumor-suppressing effects reported. Abbreviations: AE2, anion exchanger 2; Cyp2e1, cytochrome P450 Family 2 Subfamily E Member 1; Cyp2b10, cytochrome P450 Family 2 Subfamily B Member 10; DLGAP4, DLG associated protein 4; FAO, fatty acid oxidation; GSK3β, glycogen synthase kinase-3β; IGF, insulin-like growth factor; IL, interleukin; PDC-E2, E2 components of pyruvate dehydrogenase complexes; PDK1, pyruvate dehydrogenase kinase 1; PPAR, peroxisome proliferator–activated receptor; SGK1, serum/glucocorticoid regulated kinase 1.

The pathogenesis﻿ of metabolic dysfunction–associated steatotic liver disease/metabolic dysfunction–associated steatohepatitis (MASLD/MASH) is complex in multiple aspects, including lipotoxicity, insulin resistance, inflammation, and mitochondrial dysfunction.^65^ These are not simple independent events but rather interact with each other, ultimately leading to the most significant prognostic factor, liver fibrosis.^66^,^67^ PPARD can modulate MASLD pathophysiology via different mechanisms (Figure 2A). PPARD regulates gene expression involved in fatty acid oxidation in the liver, and the use of its agonist (GW501516) in diet-induced or genetic models of obesity stimulated fatty acid oxidation and improved hepatic steatosis.^45^,^68^ The selective PPARD agonist, seladelpar, ameliorated hepatic steatosis in diabetic obese mice via an autophagy-mediated pathway,^69^ and also inhibited lipotoxicity and improved NASH.^70^ Activation of PPARD in mouse models increased energy consumption, improved insulin sensitivity, and was protective against obesity and diabetes.^71^ Regarding insulin resistance, activation of PPARD has been reported to improve high-fat diet-induced insulin resistance via the IL-6/signal transducer and activator of transcription 3 pathway by increasing AMP-activated protein kinase activity in mice.^12^ In terms of inflammation, the loss of normal KCs by apoptosis and the activation of HSCs and LSECs by inflammatory cytokines such as TNF and IL-1β are thought to be one mechanism for MASLD progression.^72^ PPARD is also expressed in KCs and its activation suppressed inflammatory cytokines and ameliorated steatohepatitis in mice.^70^,^73^ Activated HSCs convert to a myofibroblast phenotype that promotes fibrosis and contributes to MASH progression, mediated by connective tissue growth factor and TGF-β.^74^ PPARD is expressed in HSCs, but its effect on fibrosis is controversial.^14^,^55^,^75^ Interestingly, PPARD expression is controlled by the circadian rhythm in mice,^76^,^77^ and treatment of MASLD/MASH by restoring circadian rhythms in hepatic metabolism is a novel approach.

#### Primary biliary cholangitis

PBC is an autoimmune cholestatic liver disease characterized by chronic inflammation of the portal area and destruction of small bile duct cells.^78^ PPARD is a potential therapeutic target for PBC in terms of bile acid secretion, anti-inflammation, immune regulation, and cell survival. The PPARD agonist seladelpar significantly reduced the bile acid precursor 7α-hydroxy-4-cholesten-3-one (C4) and total bile acids in patients with PBC.^79^,^80^ Furthermore, this suppressive effect for bile acid synthesis is dependent on induction of FGF21.^15^ PPARD agonism has also been reported to increase bile flow threefold in mice.^81^ PPAR activation reduces inflammation by suppressing activator protein 1 and NF-κB signaling in cells and mouse models.^82^ As for PPARD, it is involved in the promotion of differentiation into M2-macrophages, including KCs,^51^ and in the suppression of polarization of type 1 and type 17 helper T cells.^5^ These reports are consistent with clinical findings that PPARD agonists reduced blood IgM and high-sensitivity C-reactive protein levels in patients with PBC.^17^ In this report, the drug markedly improved pruritus, a symptom that significantly affects the quality of life of patients with PBC, and the most recent related report found that it also significantly reduced IL-31, a known pruritus-mediating cytokine.^83^ In PBC, epigenetic downregulation of anion exchanger 2 (AE2) in intrahepatic cholangiocytes causes a decrease in the protective layer of bicarbonate on the cell surface and an increase in intracellular pH. As a result, apoptosis is induced by bile acids and the released PDC-E2 sensitizes antigen-presenting cells, leading to a state of immune overload. Although PPARD expression in cholangiocytes has been reported,^40^,^41^,^42^,^43^ its role in this cell death process, which is critical for PBC pathogenesis, is unknown and is a subject for future study. A schematic of the molecular mechanism of PPARD in PBC is shown in Figure 2B.

#### Alcohol-associated liver disease

PPARD agonists prevent alcohol-associated liver disease (ALD) in mouse models. The main reasons for this include the regulation of hepatic cytochrome P450 (CYP), insulin resistance, autophagy, bile acid homeostasis, and gut-liver axis. Hepatic cytochrome P450 family 2 subfamily E member 1, an enzyme that plays an important role in ALD pathogenesis, is upregulated in *Ppard* knockout mice.^84^ On the other hand, CYP2B10, which is protective against ALD, is downregulated by *Ppard* gene ablation independent of chemically activated nuclear receptors constitutive androstane receptor, a known regulator of CYP2B.^85^ Regarding insulin resistance, intraperitoneal﻿ administration of ﻿PPARD agonist L-160, 043 to alcohol-exposed rats significantly increases the binding of insulin to IGF 1 and 2 receptors.^86^ Autophagy-mediated pathways promote fatty acid catabolism and inhibit hepatic fat accumulation by activating PPARD.^69^ Alcohol consumption suppresses autophagy and promotes apoptosis in the liver,^87^ and PPARD may improve ALD via autophagy-related pathways. In addition, the pathogenesis of ALD involves changes in bile acid homeostasis and intestinal barrier disruption.^88^ The selective PPARD agonist seladelpar reduced total bile acids in the liver and serum, and reduced ethanol-induced liver disease in mice^89^ PPARD activation by selective ligands also improved intestinal barrier function, supporting that bile acid metabolism and gut-liver axis are therapeutic targets for ALD treatment by PPARD agonists.^90^ The protective effect of intestinal barrier function is mediated by PPARD rather than PPARA in a mouse model of ALD.^91^ A schematic of the molecular mechanism of PPARD in ALD is shown in Figure 2C.

#### Hepatocellular carcinoma

The function of PPARs in HCC varies by subtype and is not fully understood. For PPARD, both tumor-promoting and tumor-suppressing effects have been described. As a tumor-promoting effect, ligand-activated PPARD induced HCC cell proliferation and invasion through the pyruvate dehydrogenase kinase 1/protein kinase B (﻿AKT)/GSK3β signaling pathway.^92^ Disc large associated protein 4 (﻿DLGAP4)﻿, which is highly expressed in HCC cell lines and tissues, increased PPARD expression and promoted HCC proliferation and metastasis.^93^ In contrast, the PPARD agonist GW501516 inhibits the proliferation of Hepa 1–6 cells.^94^ Another PPARD agonist, GW0742, suppresses HCC development in HBV transgenic mice by inducing apoptosis.^95^ More recently, PPARD negatively regulates the expression of serine/threonine-protein kinase by binding directly to oncogenes, suppressing HCC development.^96^ Furthermore, berberine, an alkaloid extracted from plants, directly activates PPARD and induces apoptosis of HCC in mice and cell line experiments.^97^ However, HCC development, proliferation, and metastasis are highly complex intersecting pathways, and the role of PPARD in HCC pathogenesis will require further study. Importantly, the 2 drugs with PPAR delta agonism, elafibranor and seladelpar, passed the required carcinogenicity studies and showed no evidence of cancer progression in any of the clinical trials discussed later. A schematic of the molecular mechanism of PPARD in HCC is shown in Figure 2D.

### Progress of clinical trials of PPAR-agonists in liver disease

Because of its diverse functions in the liver, PPAR-related compounds, mainly their agonists, are being investigated as novel therapeutic candidates in liver diseases. Table 1 summarizes the status of ongoing clinical trials of PPAR-agonists for liver diseases (selective and dual agonists acting on PPARD are indicated in bold). MASLD/MASH and PBC are the primary focus of clinical trials with PPAR-agonists, since there is only one approved pharmacological treatment for MASLD/MASH and since PBC is also in need of secondary therapies for UDCA nonresponders. This section focuses on PPARD agonists (including dual agonists) currently in clinical trials and reports their characteristics, and findings to date.

**TABLE 1:** The status of ongoing clinical trials of PPAR-agonists for liver diseases

Classification	Name	Isotype selectivity	Subject liver diseases	Phase/sample size	Identification number	Status	Combination with other drugs	Primary Endpoint
Pan-PPAR agonist	Bezafibrate	α, δ, γ	PBC	III (72)	NCT04594694	Active, not recruiting	Obeticholic acid	Change in alkaline phosphatase from baseline to Week 12
			PBC	II (60)	NCT05239468	Active, not recruiting	Obeticholic acid	Change in alkaline phosphatase from baseline to Week 12
			PBC	Observational (100)	NCT04514965	Recruiting		
	Lanifibranor	α, δ, γ	NAFLD/NASH	III (1000)	NCT04849728	Recruiting		Resolution of NASH and improvement in fibrosis
			NAFLD/NASH	II (42)	NCT05232071	Active, not recruiting	sodium-glucose cotransporter-2	Absolute change in HbA1c from baseline (Week 0) to Week 24
Dual PPAR agonist	Saroglitazar	α, γ	NAFLD/NASH	II (60)	NCT03617263	Recruiting		Change in hepatic fat content from baseline following 24 weeks of treatment as measured by MRI-PDFF
			NAFLD/NASH	II (240)	NCT05011305	Recruiting		Improvement in liver fibrosis with no increase in NAS for ballooning, inflammation or steatosis
			NAFLD/NASH	IV (1500)	NCT05872269	Recruiting		Liver stiffness measurement performed by transient elastography
			PBC	IIb/III (186)	NCT05133336	Active, not recruiting		Proportion of subjects with biochemical response based on the composite endpoints of alkaline phosphatase and total bilirubin
	Pioglitazone	α, γ	NAFLD/NASH	II (100)	NCT05254626	Active, not recruiting		Resolution of total NAS defined as absence of NASH
			NAFLD/NASH	II (166)	NCT04501406	Recruiting		Improvement of≥2 points in NAS without an increase in fibrosis stage
	Elafibranor	α, δ	PBC	III (161)	NCT04526665	Active, not recruiting		Percentage of participants with response to treatment measured by the combination levels of alkaline ﻿phosphate ﻿levels and total bilirubin
			PBC	III (450)	NCT06016842	Recruiting		Event-free survival
Selective PPAR agonist	Pemafibrate	α	PBC	II (45)	NCT06247735	Recruiting		Percent change from baseline in alkaline phosphatase
	Fenofibrate	α	PBC	III (150)	NCT05751967	Recruiting	Ursodeoxycholic acid	Percentage of patients with complete biochemical response
			PBC	II and III (104)	NCT05749822	Recruiting		Percentage of patients with biochemical response
			PBC	II and III (184)	NCT06174402	Recruiting		Percentage of patients with biochemical response
			PBC	II and III (117)	NCT06365424	Recruiting		Percentage of patients with biochemical response
	Seladelpar	δ	PBC	II and III (500)	NCT03301506	Recruiting		Treatment emergent adverse events, biochemistry and hematology results
			PBC	I (24)	NCT04950764	Recruiting		Evaluate maximum concentration of seladelpar and metabolites etc
			PBC	III (192)	NCT06051617	Recruiting		Event-free survival
			PBC	III (150)	NCT06060665	Recruiting		Proportion of subjects who achieve normalization of ﻿alkaline ﻿phosphate (≤ ULN) and≥15% decrease

Abbreviations: MRI-PDFF, magnetic resonance imaging proton density fat fraction; NAS, NAFLD activity score; PBC, primary biliary cholangitis; PPAR, peroxisome proliferator–activated receptor.

#### Dual PPAR agonists

Elafibranor (GFT-505) is a dual PPARA/PPARD agonist. As such, it is characterized by the absence of cardiovascular side effects experienced with PPARD ligands. In NASH, the efficacy of elafibranor (120 mg/d, 72 wk) was verified in a phase III clinical trial that enrolled 2157 patients mainly in Europe and the United States. Unfortunately, the study was terminated in March 2022 because it showed no significant difference compared with placebo in the primary endpoint of eliminating NASH without worsening fibrosis (GENFIT Press Release [May 11, 2020]). Currently, the primary target of evaluation for this drug is PBC. In a phase II study, 45 patients with PBC who were nonresponders to UDCA were treated with elafibranor for 12 weeks (80 mg, 120 mg). The relative change (%) from baseline to 12 weeks in serum alkaline phsophatsase, the primary endpoint, was −48.3 ± 14.8% (mean change ± SD) in the 80 mg group and −40.6 ± 17.4% in the 120 mg group, compared with +3.2 ± 14.8% in the placebo group. In addition, other markers such as GGT, IgM, and CRP were also significantly reduced in the treatment group.^98^ In terms of safety, there were no serious adverse events in the placebo and elafibranor 80 mg groups, but adverse events were reported in 2 patients in the 120 mg group. Based on these promising results, a phase III clinical trial (NCT04526665) is ongoing to evaluate the long-term efficacy and safety of the drug. According to the first report, 161 patients were randomized to elafibranor 80 mg or placebo, and the percentage of patients with a biochemical response at 52 weeks was 51% in the elafibranor group versus 4% in the placebo group (95% CI: 32–57; *p*<0.001).^16^ Based on these results, elafibranor (80 mg) was conditionally approved by the FDA and the European Medicines Agency as second-line therapy for patients with PBC nonresponding to UDCA.

#### Selective PPARD agonist

Seladelpar (MBX-8025) is a selective PPARD agonist with anti-inflammatory and cholestasis inhibitory effects. Its efficacy on the liver has already been reported from several animal studies,^14^,^15^ and there are also reports of clinical trials in MASLD/MASH and PBC. A phase II clinical trial initiated in June 2018 with 181 patients with NASH showed no significant MRI-proton density fat fraction differences between the placebo and treatment groups at 12 weeks. On the other hand, preclinical data show beneficial effects on fatty liver,^69^,^70^ and further studies are needed to translate PPARD biology from rodents to humans. More clinical trials have been conducted in the area of PBC.^79^,^99^,^100^ Very recently the results of a phase III trial, the RESPONSE study, were published.^17^ In this phase 3, double-blind, randomized, placebo-controlled trial, 193 patients with PBC with UDCA nonresponse or unacceptable adverse events were randomized in a 2:1 ratio to receive oral seladelpar (10 mg/d) or placebo. The primary endpoint was biochemical response at 12 weeks, defined as an alkaline phsophatsase level <1.67 times the upper limit of the normal range, a decrease of at least 15% from baseline, and a normal total bilirubin level. As a result, a greater percentage of the patients in the seladelpar group than in the placebo group had a biochemical response (61.7% vs. 20.0%; difference, 41.7 percentage points; 95% CI: 27.7–53.4, *p*<0.001). Based on these results, seladelpar (10 mg) was conditionally approved by the FDA as second-line therapy for patients with PBC refractory to UDCA. Several clinical trials are still ongoing to evaluate the efficacy and safety of seladelpar for PBC (Table 1). In 2017, a large cohort open-label Phase 3 study (ASSURE, NCT03301506) was initiated to evaluate the safety of seladelpar (5 and 10 mg). Patients enrolled in the aforementioned RESPONSE study are also eligible to participate, and 500 patients will be evaluated for adverse events for up to 60 months. Separate studies are underway to evaluate the safety of seladelpar 10 mg in patients with PBC with compensated cirrhosis (NCT04950764, NCT06051617).

## CONCLUSIONS AND PERSPECTIVE

The results of several clinical trials have shown that PPARD is a promising new therapeutic target for liver diseases, especially PBC and MASLD/MASH. Moreover, PPAR studies have increasingly clarified its specific liver functions. There is no doubt that this molecule plays an important role in liver physiology and disease pathogenesis, regulating not only lipid, glucose, and bile acid metabolism, but also alcohol metabolism, inflammation, fibrosis, and carcinogenesis, among other mechanisms. Interesting facts have also emerged regarding distribution in the liver, function in each liver cell type, sex differences, and circadian rhythms. On the other hand, there is still insufficient human data to support these facts, which is the next challenge in the era of personalized medicine. We believe that both clinical and comprehensive molecular biological approaches related to PPARD can lead to better clinical outcomes for patients with liver disease.
